# *In vitro* Assessment of Solar Filters for Erythropoietic Protoporphyria in the Action Spectrum of Protoporphyrin IX

**DOI:** 10.3389/fmed.2021.796884

**Published:** 2021-12-20

**Authors:** Alvise Sernicola, Elena Cama, Maria Guglielmina Pelizzo, Enrico Tessarolo, Annamaria Nicolli, Giulia Viero, Mauro Alaibac

**Affiliations:** ^1^Dermatology Unit, Department of Medicine (DIMED), University of Padova, Padua, Italy; ^2^Department of Information Engineering, University of Padova, Padua, Italy; ^3^Institute for Photonics and Nanotechnologies, National Research Council of Italy, Padua, Italy; ^4^Department of Cardiac, Thoracic, Vascular Sciences and Public Health (DCTV), University of Padova, Padua, Italy

**Keywords:** erythropoietic protoporphyria, photoprotection, protoporphyrin IX, sunscreen, cutaneous porphyria, Photodermatosis

## Abstract

**Introduction:** Subjects with erythropoietic protoporphyria rely on broad-spectrum sunscreens with high sun protection factor, which is not informative on efficacy in the absorption spectrum of protoporphyrin IX, spanning visible radiation and peaking around 408 nm. Photoactivation of protoporphyrin IX is responsible for painful skin photosensitivity in erythropoietic protoporphyria.

The authors assessed the protective efficacy of six sunscreens *in vitro* in the absorption spectrum of protoporphyrin IX.

**Method:** Transmittance measurements were performed in the 300–850 nm wavelengths on samples of six photoprotective products applied to polymethyl methacrylate plates. Porphyrin protection factor was calculated in the 300–700 nm region to provide a measurement for the efficacy of each product based on the action spectrum of protoporphyrin IX.

**Results:** Product A showed the highest porphyrin protection factor among tested products with a median value of 4.22. Product A is a sunscreen containing organic filters, titanium dioxide and synthetic iron oxides, pigmentary grade active ingredients that absorb visible radiation. Other products showed inefficient protection in the visible, with transmittance between 75 and 95% at 500 nm. The low porphyrin protection factor of inorganic filter product B was attributed to particle micronization, as declared by the manufacturer.

**Conclusion:** Adding porphyrin protection factor to sunscreen labeling could help patients with erythropoietic protoporphyria and other photosensitivity disorders identify products tailored on their specific needs. The development of sunscreens providing protection from visible radiation and excellent cosmetical tolerability could improve the lifestyle of patients with erythropoietic protoporphyria.

## Introduction

Erythropoietic protoporphyria (EPP) is a cutaneous porphyria resulting from loss-of-function mutations in the ferrochelatase gene (*FECH*) with autosomal recessive Mendelian inheritance (1). Alternatively, gain-of-function mutations in aminolevulinic acid synthase gene (*ALAS2*) are responsible for the closely related phenotype termed X-linked protoporphyria (XLP) with X-linked inheritance (2). Additional mutations have been reported (3) and EPP can also develop *de novo* in the context of myeloproliferative disorders (4, 5). Most commonly, EPP phenotype results from the inheritance of a common hypomorphic *FECH* variant, IVS3-48T/C, together with a loss-of-function allele of the same gene (6). This leads to insufficient activity of the FECH enzyme which inserts iron or zinc in the protoporphyrin IX ring, finalizing heme formation in the inner mitochondrial membrane (1). The result is excessive metal-free protoporphyrin accumulation occurring in plasma and erythrocytes. Metal-free protoporphyrin diffuses to plasma from erythrocytes, where it is bound to hemoglobin and released following light irradiation, and directly from reticulocytes in the bone marrow (7–9). Metal-free protoporphyrin accumulates in plasma contributing to photosensitivity and is bound to albumin, which provides the only excretion pathway for this water-insoluble porphyrin through the liver (9–11). Hydrophobic protoporphyrin deposits in lipidic layers of cell membranes (12) in the skin, where acute painful non-blistering photosensitivity develops. Liver damage is an uncommon finding potentially complicating EPP in as few as 5% of cases (13, 14). However, the risk of gallstones containing water-insoluble protoporphyrin is increased to up to 8% of patients (14). Excluding patients who develop protoporphyria-related hepatopathy, the risk for which cannot be accurately predicted (15), life expectancy in EPP is not shortened compared to the general population. This leads to lasting impairment on employment and lifestyle due to the need to avoid sunlight. Onset of photosensitivity in EPP occurs early in childhood, at an average before the age of four (16) and with no difference between sexes. However, diagnosis is commonly delayed to an average of 13 years from the first symptoms (16), during which undiagnosed children and adults endure unexplained pain and withdrawal from daily social activities. Following exposure to sunlight or fluorescent light sources, severe skin pain with or without signs of erythema, edema, or blanching, develops acutely after a median of 20 min and resolves after a median of 3 days (14) leaving little to no residual skin damage. Chronic changes may affect the skin on the knuckles and back of hands and on the face in patients subject to repeated light exposure (17–19). Physical findings are related to individual differences in pigmentation, degree of sun exposure and reportedly to a priming phenomenon (14). The severity of symptoms is also dependent on the level of erythrocyte protoporphyrin, for which no corrective intervention is available. Protection from sunlight exposure is the main strategy to manage the symptoms of EPP and to limit impairment of educational and employment opportunities in these patients. Therapies to increase light tolerance include subcutaneous afamelanotide and oral beta-carotene. Synthetic alpha-melanocyte stimulating hormone afamelanotide has been available in Europe since 2014 as a 16 mg subcutaneous implant administered every 2 months 3 times a year to adult patients (20), and effectively improves sunlight tolerance allowing patients to engage in activities that would previously be avoided (21). However, evidence supporting its use in pediatric patients is lacking, as is safety data in pregnancy or in liver and kidney disease (22). Use of beta-carotene proved beneficial in some patients (23, 24); however, overall evidence is inadequate and results of a randomized controlled trial showed no improvement in light tolerance compared to placebo (25). Subjects with EPP require photoprotection in the UV-A and visible light regions, where the absorbance spectrum of protoporphyrin lies (26). The efficacy of commercially available sunscreens is commonly assessed in the UV-B and UV-A bandwidth; however, their protective effect against visible light is generally not significant and poorly characterized. A photoprotective index named porphyrin protection factor (PPF) was recently introduced to measure the *in vitro* efficacy of photoprotective products based on the absorbance of protoporphyrin IX in the 300–450 nm wavelength region (27). As current commercial sunscreens do not significantly absorb in this region, specific effective topical photoprotection is a current area of unmet need in EPP.

The present study aimed at assessing the *in vitro* protective efficacy of six sunscreens by calculating PPF, as previously described in the 300–450 nm region and extended to the 300–700 nm wavelengths to include different absorption peaks of protoporphyrin IX: an intense Soret band centered around 406 nm and four weaker Q bands in the visible range (26).

## Method

### Tested Photoprotective Products

Six sunscreens were independently selected by the investigators among commercially available products labeled with high SPF and wide-spectrum protection from radiations including UV-A (Table 1).

**Table 1:** Labeling information with SPF, filter type and results of PPFs measurements in the 300–700 nm wavelengths of the six tested solar filters, expressed as median [min;max]. COV, coefficient of variation.

**Product (label SPF)**	**Principal active components**	**PPF 300-700 nm**	**COV**
**A (50+)**	•Octocrylene, •Methylene bis-benzotriazolyl tetramethylbutylphenol [nano], •Butyl methoxydibenzoylmethane, •Titanium dioxide (ci 77891), •Bis-ethylhexyloxyphenol methoxyphenyl triazine, •Iron oxides (CI 77492, 77491, 77499).	4.22 [3.34;4.78]	0.1226
**B (30)**	•Zinc oxide (nano), •Titanium dioxide (nano).	1.73 [1.66;1.75]	0.0270
**C (100+)**	•Ethylhexyl methoxycinnamate, •Diethylamino hydroxybenzoyl hexyl benzoate, •Octocrylene, •Ethylhexyl triazone, •Titanium dioxide (nano), •Bis-ethylhexyloxyphenol methoxyphenyl triazine.	1.86 [1.76;1.92]	0.0365
**D (50+)**	•Ethylhexyl methoxycinnamate, •Methylene bis-benzotriazolyl tetramethylbutylphenol (nano), •Bis-ethylhexyloxyphenol methoxyphenyl triazine, •Ethylhexyl salicylate, •Diethylamino hydroxybenzoyl hexyl benzoate.	1.82 [1.73;1.84]	0.0259
**E (50+)**	•Octocrylene, •Ethylhexyl salicylate, •Butyl methoxydibenzoylmethane, •Ethylhexyl triazone, •Bis-ethylhexyloxyphenol methoxyphenyl triazine.	1.65 [1.61;1.68]	0.0171
**F (50+)**	•Bis-ethylhexyloxyphenol methoxyphenyl triazine, •Ethylhexyl triazone, •Diethylamino hydroxybenzoyl hexyl benzoate, •Diethylhexyl butamido triazone.	1.53 [1.52;1.59]	0.0190

### *In vitro* Substrates

Samples were placed on polymethyl methacrylate (PMMA) square plates (Schönberg GmBH & Co, Hamburg, Germany) sized 50 × 50 × 2.5 mm and with a rugosity around 6 μm. PMMA plates are appropriate substrates for *in vitro* assessments of UV-A blockers according to COLIPA (European Cosmetics Trade Association) guidelines (28) and are used in previous published studies (27). Five samples of each product were each applied to the upper surface of a PMMA plate using a positive-displacement dispenser (Multipette; Eppendorf AG, Hamburg, Germany). Samples were weighted using a precision scale (Δ*g* = ± 0.1 mg) to assess correct application rate and consistent distribution among samples with a surface density of 1 mg/cm^2^. Amounts of 1 mg/cm^2^ applied on PMMA plates showed good correlation between *in vitro* and *in vivo* SPF in previous studies (29). For the present investigation, a single rate of application was chosen to allow comparison between the absorbance curves of different samples *in vitro*. According to the authors' previous experimental results obtained for PMMA plates with 1 mg/cm^2^ of product application, the correlation between *in vitro* measurements and *in vivo* SPF does not yield a 1:1 correspondence but can be described with a regression line (30, 31). For this reason, evaluation of *in vitro* SPF values remained beyond the scope of this study.

### Transmittance Measurements

Transmittance measurements were performed in the 300–850 nm range using an integrating sphere spectrophotometer (Cary 5000; Varian Medical Systems, Inc., Palo Alto, CA, U.S.) with photomultiplier tube R928 (Hamamatsu Photonics K.K., Hamamatsu City, Japan). Single measure statistical error for this equipment is reported around 1%. Transmittance of substrates (T%) was expressed as percentage of the baseline transmittance of each PMMA plate before application of each sample.

### Calculation of *in vitro* Protection Factor

*In vitro* efficacy of each sunscreen was expressed using porphyrin protection factor (PPF), as previously defined in the literature as ratio of two integrals calculated in the spectral range of 300–450 nm (27) and as modified by the authors in the range of 300–700 nm to include measurement of protective effect against damage from visible light:


(1) $$PPF{=}^{\int_{\lambda =300}^{\lambda =700}PP(\lambda )I(\lambda )d\lambda }\text{​​}╱\text{​}{\text{​}}_{\int_{\lambda =300}^{\lambda =700}PP(\lambda )I(\lambda )10^{-A(\lambda )}d\lambda }$$

Calculations are based on the absorbance *A*(λ) retrieved by the transmittance value, and on action spectrum PP(λ) of protoporphyrin IX and on the standard solar spectral irradiance *I*(λ) with air mass of 1.5 G (32).

### Statistical Analysis

Analyses were performed using the SAS 9.4 package (SAS Institute Inc., Cary, NC, USA) on Windows. The continuous variable PPF was presented as median and range of the measurements for the substrate of each sample. Comparison of PPF between the six different samples was performed using the Kruskal-Wallis test, a non-parametric test for multiple independent samples. When significance was found from this test, the Dwass-Steel-Critchlow-Fligner test, a two-sided non-parametric procedure, was used to determine which groups were different. Results were considered significant at *p* < 0.05. Differences between medians estimated using the Hodges-Lehman statistics were presented as 95% confidence intervals.

## Results

Product A, a physical barrier cream, resulted in a median PPF value of 4.22 [range = 3.34–4.78], the highest among tested products. *In vitro* PPFs for the remaining samples are reported in Table 1 (=median [range]). The Kruskal-Wallis test on the six samples showed significance (*p* < 0.0001) and PPFs were then compared pairwise between products demonstrating a statistically significant difference (*p* < 0.05) between medians of product A and each of the five other samples (95% confidence interval): vs. product B (1.68;3.03), vs. C (1.77;2.84), vs. D (1.61;2.94), vs. E (2.00;2.98), vs. F (1.82;3.19).

## Discussion

**Figure 1:**
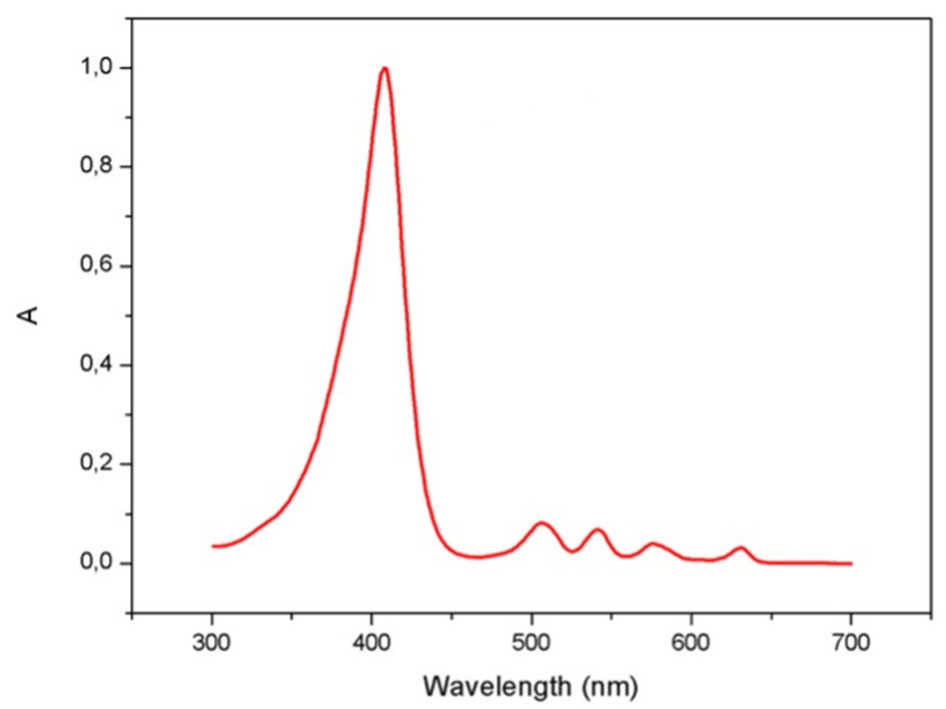
Absorbance (A) spectrum of protoporphyrin IX measured in the range 300–700 nm.

Porphyrins are photoactive compounds that cause damage to biological molecules, following activation by light and transfer of energy to highly oxidizing oxygen species (12, 33–35). Protoporphyrin IX absorption occurs between 320 and 595 nm and peaks in the 400–420 nm wavelength, known as the Soret band for porphyrins, which lies in the visible range close to UV-A light (36) (Figure 1). Poor quality of life in EPP patients is related to sunlight avoidance that, though effectively preventing symptoms, restricts recreational and productive activities (37). The appropriate management of this condition must therefore focus on providing strategies to maximize light tolerance and allow engagement in normal daily activities. Topical sunscreens, combined with hats and protective clothing when outdoors and lifestyle adaptations (38, 39), are a key photoprotective intervention in EPP.

Commercially available products contain organic and inorganic filters that are well characterized according to their protection from UV-B and UV-A radiation (40, 41) but lack clear efficacy against visible light, which is the culprit in EPP (42). Subjects with EPP commonly rely on broad-spectrum sunscreens with SPF of 30 or higher. Conventional SPF, however, is not informative on the protective efficacy in the visible radiation and especially around 408 nm, where protoporphyrin IX has its highest peak of absorption. For this reason, PPF was previously introduced as an index independent of SPF and PFA.

In 1991, an *in vitro* study first investigated the protection efficacy in UV-A and visible wavelengths of an inorganic filter with 20% zinc oxide combined with pigmented iron oxide (43). A later study proposed a photosensitivity protection factor, based on the conventional SPF formula adjusted to include wavelengths up to 600 nm, to assess inorganic filters containing 4% titanium dioxide and 5% zinc oxide (42). In 2017, Teramura et al. further adapted this index and introduced PPF calculated in the spectral range of 300–450 nm, which is most harmful for individuals with EPP. The authors demonstrated high PPF values in a make-up base emulsion and liquid and powder foundations containing colored and absorbing pigment iron oxide (27). Moreover, the products were tested for 6 months in EPP patients showing efficacy over 78% and no adverse reactions (27).

**Figure 2:**
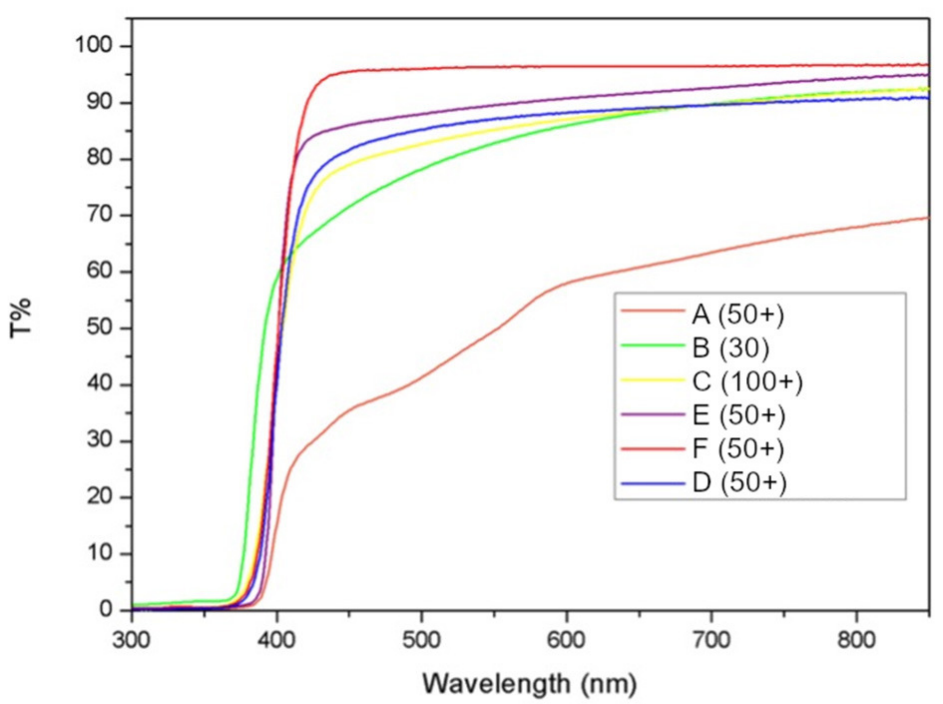
Measured transmittance for each of the six tested products. Transmittance of substrates is calculated as percent of the baseline transmittance of each PMMA plate before sample application (T%).

The photoprotective products tested in this study include organic and inorganic filters devised to shield UV-B and UV-A radiations, that are the main culprits of erythema in generally healthy subjects and show transmittance close to 0% in these wavelengths. Protection is inefficient in the visible, with transmittance for products B-F between 75 and 95% at 500 nm. On the other side, product A transmitted <40% of radiation at the same frequency (Figure 2).

Our preliminary results show that product A has the highest PPF among tested products with a median value of 4.22. Product A is a sunscreen containing multiple organic UV filters, inorganic filter titanium dioxide and synthetic iron oxides. The latter are colored pigments that absorb radiation in the range 300–700 nm and, together with inorganic filters titanium dioxide and zinc oxide, justify the performance of this sunscreen (44). Product B scored a poor PPF attributed to micronization—declared by the manufacturer—of inorganic filters zinc oxide and titanium dioxide that shifts protection away from visible wavelengths and toward UV-B and UV-A (45).

**Figure 3:**
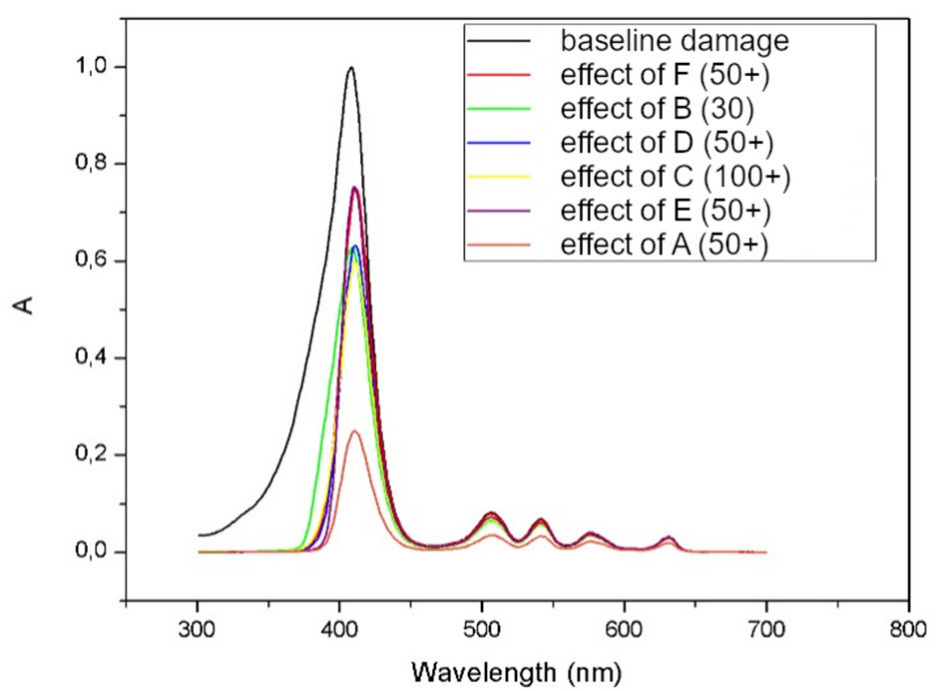
Absorbance (A) spectrum of protoporphyrin IX before (black line represents baseline damage) and after subtracting the action spectrums of each of six tested products (lower red line represents protection of product A).

The main limitation to the present study is the absence of a proper skin surrogate; though PMMA is a widely used substrate, transmittance measures can be affected by uneven sample distribution and porosity on the material's surface. The authors prepared five samples of each product to improve reproducibility of PPF. According to the preliminary results of this study, a sunscreen containing organic and inorganic filters with pigmented iron oxides, such as product A, showed the highest PPF, with a median of 4,22 among photoprotective products tested *in vitro* (Figure 3).

In conclusion, PPF is indicative of sunscreen protection *in vitro* and could help patients with EPP, as well as those with other disorders of photosensitivity, identify products tailored on their specific needs (45). Effective protection in real life requires correct use of sunscreens with reapplication of product every 2 h of sun exposure. Finally, future research in skin pharmacology could provide filters that significantly absorb visible radiation while improving cosmetical tolerability for patients.

## Data Availability Statement

The raw data supporting the conclusions of this article will be made available by the authors without undue reservation.

## Author Contributions

AS was responsible for writing the original draft and for visualization. EC was responsible for investigation and for writing the original draft. MP was responsible for project administration and investigation. ET was responsible for methodology and investigation. AN was responsible for investigation and formal analysis. GV was responsible for investigation and for writing the original draft. MA was responsible for conceptualization and supervision. All authors contributed to review and editing of the manuscript.

## Conflict of Interest

The authors declare that the research was conducted in the absence of any commercial or financial relationships that could be construed as a potential conflict of interest.

## Publisher's Note

All claims expressed in this article are solely those of the authors and do not necessarily represent those of their affiliated organizations, or those of the publisher, the editors and the reviewers. Any product that may be evaluated in this article, or claim that may be made by its manufacturer, is not guaranteed or endorsed by the publisher.
